# The surface texture properties after electrical discharge machining with negative polarity using graphite electrodes of different grain sizes

**DOI:** 10.1038/s41598-025-33799-6

**Published:** 2025-12-28

**Authors:** Rafał Nowicki, Rafał Świercz

**Affiliations:** Institute of Manufacturing Technology, Faculty of Mechanical and Industrial Technology, Warsaw University of Technology, Narbutta 85, 02-524 Warsaw, Poland

**Keywords:** Engineering, Materials science

## Abstract

Electrical discharge machining (EDM) is a precise, unconventional manufacturing technique widely used for machining difficult-to-cut materials. Hastelloy C-22, a nickel-chromium alloy, presents significant challenges for conventional machining due to its high hardness and melting point. Graphite electrodes are commonly employed in EDM because of their favourable technological and economic properties. In this study, two commercially available POCO graphite electrodes with different grain sizes were used: 1 $\upmu$m (AF-5) and 10 $\upmu$m (S-180). The investigation focused on the effect of graphite grain size and EDM electrical parameters on the surface roughness of Hastelloy C-22 under negative polarity, which is often selected to improve material removal efficiency and surface finish. Surface topography was quantitatively characterised using *Sa*, *Sz*, *St*, *Sds*, *Sdr*, and *Sfd* parameters. The results indicate that surface roughness is primarily determined by electrical parameters (current intensity, discharge duration, and time interval), with the influence of graphite grain size being relatively minor. Grain size affects tool wear and the stability of electrical discharges, which can induce surface irregularities. Statistical models were developed to predict surface roughness based on EDM parameters, providing guidance for optimising the machining of Hastelloy C-22 with different graphite electrodes.

## Introduction

Electrical discharge machining (EDM) is an advanced manufacturing technique commonly employed to precisely shape intricate three-dimensional parts made of conductive materials. In this type of machining, material removal occurs through electrical discharges between two electrodes connected to a pulse generator that provides current. One electrode is the workpiece, and the other is the tool electrode. During operation, two different polarity configurations are possible: positive and negative^1,2^ . When set to positive polarity, the tool electrode is connected to the positive terminal of the power supply, making the workpiece the cathode. In contrast, in negative polarity, the tool electrode is connected to the negative terminal, with the workpiece acting as the anode^3–8^.

EDM has been a widely used manufacturing technology for many years. Thanks to the non-contact processing method, it is possible to machine both electrically conductive materials and semiconductors with a conductivity more excellent than 0.01 S/cm, regardless of their mechanical properties. The material removal mechanism makes EDM a rational alternative to accurately machining tough materials with complex geometric shapes^9^. The EDM process is widely implemented to manufacture components that are hard to machine by milling or turning, such as hot stamping dies, injection molds for plastic, and parts used in the surgical and aerospace industries. Electrical discharge machining has found application in new fields of science, such as medicine, surgery, optics, and nuclear energy, as well as research and development areas in the automotive industry^10–13^.

Electrical discharges induce localized melting and vaporization in both the workpiece and the tool electrode material. Throughout this process, numerous electrical discharges occur, with the energy of these discharges shaping the distinct surface characteristics of the workpiece^14–17^. The resulting surface topography is formed by the accumulation of overlapping craters generated by individual discharges. The surface texture is influenced by various physical phenomena that take place during the EDM machining process. The thermal characteristics of the electrical discharge induce several modifications to the morphological characteristics of the machined surface^18,19^. A thorough examination of the surface texture is crucial for understanding the operational performance and tribological properties of the workpiece. The properties of the finished surface are influenced by various EDM parameters, including discharge voltage, discharge time, discharge current, and time interval, as well as the type of dielectric medium used and the electrode material used^20–30^.

Graphite has become more widely used as a tool electrode material in modern industrial processes because of its advantageous technical and economic properties. In recent years, several studies have compared the performance of different electrode materials in EDM. Graphite, copper, copper–tungsten, and composite electrodes exhibit different characteristics in terms of electrical conductivity, wear rate, machining stability, and surface finish of the machined workpiece. Among these, graphite electrodes are often valued for their high isotropy, good machinability, resistance to thermal shock, and stable spark generation, which under various operating conditions produce competitive or superior material removal rates (MRR) and acceptable tool wear ratios - compared to metallic electrodes^31–34^. Meanwhile, copper, copper‑tungsten or composite electrodes can offer advantages when a very fine surface finish or minimal electrode wear is required, especially for hard or carbide workpieces. Such comparative studies provide a broader context for understanding the specific behaviour of graphite electrodes in EDM processes and justify focusing on graphite (and in particular on grain size variation within graphite) as the subject of this investigation.

In the market, graphite electrodes are classified according to their grain size, which significantly affects the results of EDM processes^1^. Contemporary scientific research has not yet explored the effect of the grain size of graphite electrodes in the EDM process on the surface texture condition of difficult-to-machine alloys based on nickel and chromium. Torres^35^ investigated how the polarity of graphite electrodes affects the surface integrity of Inconel 600. The study revealed that in terms of surface roughness metrics, using a positive polarity yields better results when a high-quality surface finish is desired. Additionally, the analysis identified current intensity and pulse duration as the most critical parameters influencing the process outcomes. Selvarajan^30^ and Sonker^32^ examined the effect of electrode material on machining performance, finding that both the material removal rate and surface roughness tend to be greater when utilising a graphite electrode in comparison to a copper electrode. Lamba^36^ performed experimental studies on the machining of EN31 steel using copper and graphite electrodes within an abrasive mixed rotary tool EDM. The results indicated that the graphite electrode yielded superior process efficiency and produced a smoother surface finish under identical operational parameters. Amorim^37^ examined the effects of electrical parameters on the sinking EDM process applied to Ti6Al4V alloy, utilising specialised graphite electrodes with particle sizes of 3, 10, and 15 $\upmu$m. The findings indicated that the optimal outcomes in terms of material removal rate, surface finish of the workpiece, and tool wear were achieved with a graphite electrode featuring a 10  $\upmu$m particle size and negative polarity. Nowicki^38^ observed that during EDM machining with positive polarity, a graphite electrode with a grain size of 1  $\upmu$m provided lower surface roughness on the workpiece than a graphite electrode with a grain size of 10 $\upmu$m. It was observed that under EDM finishing conditions, the graphite electrode grains reproduce their microgeometry on the machined surface.

Negative polarity was selected in this study because it is commonly employed in EDM to enhance material removal efficiency and improve surface finish, especially for difficult-to-machine nickel-chromium alloys. This study investigates the impact of POCO graphite electrode grain size and electrical parameters on the surface roughness of Hastelloy C-22 after electrical discharge machining. Hastelloy C-22 is a nickel-chromium alloy that presents significant machining challenges with traditional methods of manufacturing due to its elevated melting point and hardness. Consequently, electrical discharge machining is often employed for processing such materials. In current scientific publications, limited research has been conducted on the analysis of the surface texture complexity of Hastelloy C-22 alloy after electrical discharge machining with graphite electrodes of various grain sizes. Therefore, this research presents a novel approach. The findings can substantially enhance understanding of erosion-based machining with POCO graphite electrodes and may be implemented in advanced erosion machining facilities. Detailed information on the influence of the grain size of graphite electrodes and the electrical parameters of the EDM process on the material removal efficiency and the wear of the tool electrode was presented in previous studies^38,39^.

## Materials and method

The purpose of the experimental investigation was to determine the effect of the grain size of POCO graphite electrodes and variable electrical parameters on the surface roughness of Hastelloy C-22 alloy after EDM utilising negative polarisation. Two graphite electrodes with significantly different grain sizes were used: 1  $\upmu$m and 10  $\upmu$m (S-180) to verify whether the size of these particles determines the surface texture after EDM. Surface roughness analysis was performed based on the measured height (*Sa, Sz, St*) and hybrid (*Sds, Sdr, Sfd*) roughness parameters. The research methodology is described in detail in the following subsections.

### Machined material

The reference material used for the electrical discharge machining process was Hastelloy C-22 alloy. This selection was made due to the challenges encountered when machining this alloy through traditional techniques, as well as its extensive use in various industries. To date, there is a lack of scientific literature describing advanced machining techniques for Hastelloy C-22 alloy utilising highly isotropic electrodes within the EDM process. The Hastelloy C-22 alloy, due to its high content of chromium, molybdenum, and tungsten, exhibits exceptional resistance to oxidation, crevice corrosion, and stress corrosion cracking. It also demonstrates particular resistance to chloride-induced pitting. Furthermore, this alloy shows high resistance to sulfuric acid, hydrochloric acid, acetic acid, chlorine gas, seawater, brines, and various other aggressive chemical environments, both organic and inorganic, that can pose challenges during operation. Hastelloy C-22 also features excellent weldability and suitability for cold forming; however, it is challenging to machine using conventional methods of manufacturing. During machining, significant temperatures are generated in the cutting zone, resulting in increased material hardness and accelerated tool wear. Consequently, developing specialised electro-discharge machining (EDM) techniques for this alloy is justified, as they enable effective machining regardless of the mechanical properties of the material. Selected physical parameters of this alloy are presented in Table 1^40,41^.

**Table 1:** Physical properties of Hastelloy C-22 alloy.

Property	Value
Density [g/cm^3^]	8.61
Melting temperature range [$^\circ$C]	1351–1387
Specific heat [J/kg${\cdot }^\circ$C]	381
Resistance [$\mu \Omega \cdot \textrm{m}$]	1.215
Young’s modulus (dynamic) [GPa]	209
Coefficient of expansion (21-193$^\circ$C) [ $\upmu$m/m${\cdot }^\circ$C]	12.42

### Tool electrode material

Two types of graphite electrodes manufactured by POCO were selected for the experiments: AF-5 and S-180. These graphite materials differ in grain size. AF-5 graphite has a grain size of 1  $\upmu$m, while S-180 graphite has a grain size of 10  $\upmu$m. The graphite electrodes used in this study belong to commercially available isotropic POCO graphite grades, which are widely applied in industrial EDM. The POCO product line includes representative grain sizes of 1  $\upmu$m, 5  $\upmu$m, and 10  $\upmu$m. Preliminary screening tests carried out on the intermediate 5  $\upmu$m grade showed that its behaviour was intermediate between the finest and coarsest graphites and did not introduce qualitatively different discharge characteristics or surface formation mechanisms. For this reason, the present study focuses on the two extreme grain sizes (AF-5 and S-180), which provide the highest microstructural contrast and enable a clearer identification of how graphite morphology affects EDM behaviour and surface stereometry. This selection also prevents unnecessary expansion of the statistical experimental matrix while preserving mechanistic interpretability. The use of these electrode types in the study enabled a comprehensive comparison of the influence of particle size on the surface texture of Hastelloy C-22 alloy after electrical discharge machining. Detailed physical and mechanical parameters of the graphite materials are presented in Table 2.

**Table 2:** Comparison of properties between graphite S-180 and graphite AF-5.

Property	Graphite S-180	Graphite AF-5
Average particle size ( $\upmu$m)	10	1
Electrical resistivity [$\mu \Omega \cdot \textrm{m}$]	13	21.6
Apparent density (g/cm^3^)	1.78	1.8
Flexural strength (MPa)	58	117
Shore hardness	66	87

### Research method

The experimental EDM tests were conducted using a Form 2-LC ZNC EDM machine manufactured by Charmilles. The machine is equipped with an isoenergetic pulse transistor generator capable of varying the discharge current (${I}^{\hbox {c}}$), discharge time (${t}_{\hbox {on}}$), and time interval between pulses (${t}_{\hbox {off}}$).

The workpiece material used in the experimental tests was Hastelloy C-22 alloy. Samples of the alloy were prepared with dimensions of 12 mm $\times$ 2.5 mm in height. Two types of graphite electrode materials from POCO were employed: AF-5 (1 $\upmu$m particle size) and S-180 (10 $\upmu$m particle size). The electrodes had geometric dimensions of 12 x 12 x 25 mm. Prior to each experiment, both the sample and the electrodes were lapped and polished to ensure surface uniformity. The samples were eroded to a depth of 0.5 mm with a tool electrode.

A free-kinematics EDM machining model was utilised for the study, in which the tool electrode interacted solely with its frontal surface, with no lateral gaps present. This model was adopted to eliminate the influence of variations in EDM die-sinking conditions as the machining depth changed. During the EDM process, the tool electrode and the workpiece were fully immersed in a liquid dielectric – EDM fluid 108MP-SE kerosene.

**Fig. 1:**
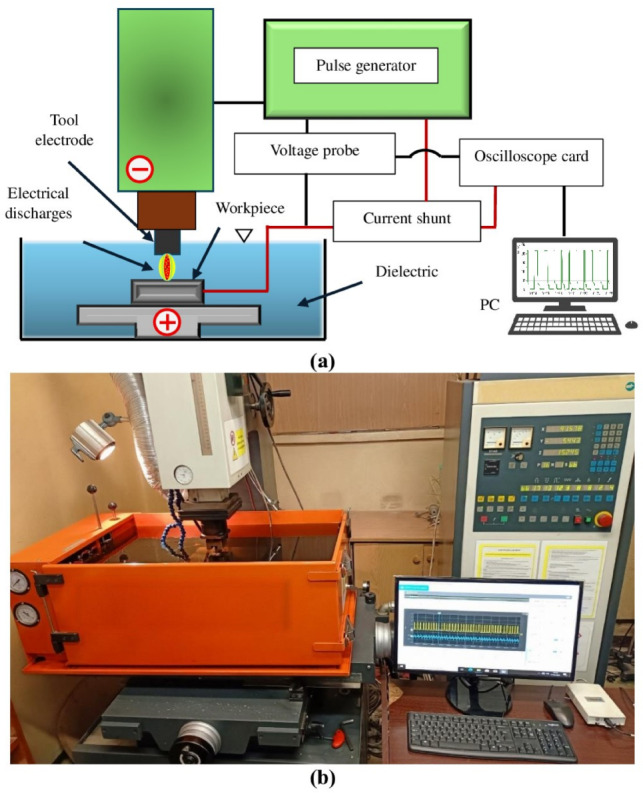
Experimental setup used for the EDM process: (**a**) schematic diagram of the test stand, showing the tool and workpiece electrodes, pulse generator, power supply, measurement instruments, DAQ card, and computer; (**b**) photograph of the EDM test stand, including the power supply and measurement circuit.

An electrical measurement circuit was employed to record the actual current-voltage characteristics. This circuit comprised a voltage probe, which measured the potential difference between the cathode and anode, and a non-inductive current shunt, which indirectly determined the discharge current based on the voltage drop across its resistance. The measurement setup allowed for real-time monitoring and control of the electrical parameters during the experiments. The experimental setup is shown in Fig. 1, which combines a photograph of the EDM machine and a schematic diagram of the process to clarify the arrangement of equipment and measurement instrumentation.

### Preliminary manufacturing test

The preliminary examination aimed to establish a range of stable parameters for semi-finishing and finishing EDM processes, which would be employed in the planned experiment to minimise surface roughness after EDM machining with graphite electrodes. For this purpose, a measuring system was used to determine the actual values of the discharge voltage (${U}_{\hbox {c}}$), discharge current (${I}_{\hbox {c}}$), discharge time (${t}_{\hbox {on}}$), and the time interval between pulses ($\textit{t}_\textrm{off}$).

The results indicated that long discharge times (${t}_{\hbox {on}}$) combined with short time intervals between pulses (${t}_{\hbox {off}}$) led to non-uniform electrical discharges. These discharges caused short-circuit pulses that damaged the workpiece surface, primarily due to excessive accumulation of machining debris within the inter-electrode gap. Among the parameters studied, the discharge current (${I}_{\hbox {c}}$) had the most significant impact on surface roughness. An increase in ${I}_{\hbox {c}}$ resulted in a greater volume of material removal, thereby enlarging the depth and diameter of the craters on the machined surface.

The relationship between discharge time (${t}_{\hbox {on}}$) and the discharge current (${I}_{\hbox {c}}$) was found to be particularly critical. At high current intensities, a short discharge duration (${t}_{\hbox {on}}$) prevented the current from reaching the set value within the gap. This phenomenon is attributed to an imbalance between the discharge current (${I}_{\hbox {c}}$) and its duration (${t}_{\hbox {on}}$). Elevated peak currents often lead to a prolonged rise time, which, combined with short pulse durations and high dielectric resistance, can hinder the ability of the machine to attain the desired current levels. Conversely, excessively short time intervals (${t}_{\hbox {off}}$) impede the removal of machining debris and detached graphite particles from the gap, increasing the risk of short-circuit discharges. During preliminary tests, it was observed that the lowest surface roughness values were achieved when machining was performed with low electrical discharge energy, specifically at low current intensity (${I}_{\hbox {c}}$) and short electrical discharge duration (${t}_{\hbox {on}}$). The most stable electrical discharge waveforms were recorded when the interval between pulses exceeded the duration of the electrical discharge.

### Experimental plan

The examination of the discharge current and voltage waveforms facilitated the identification of stable electrical parameters, which were subsequently employed for the objectives of this research. This parameter range is applicable to both finishing and semi-finishing the machining of Hastelloy C-22, employing POCO graphite electrodes.

The experimental setup involved a current intensity (${I}_{\hbox {c}}$) varying within the range of 1.7 to 5 A. The duration of the electrical discharge (${t}_{\hbox {on}}$) was controlled between 8 and 55 microseconds. The time interval separating individual pulses (${t}_{\hbox {off}}$) was adjustable from 6 to 75 microseconds. The system was powered by an open circuit voltage (${U}_{0}$) of 230 V, while the discharge voltage (${U}_{\hbox {c}}$) was maintained at 26 V. The polarity of the tool electrode was set to negative, designating the workpiece as the anode and the tool electrode as the cathode. Preliminary tests were conducted to establish a range of stable machining conditions suitable for semi-finishing and finishing processes with graphite electrodes. A measuring system was used to record the actual discharge voltage, current, pulse duration, and time interval between pulses. These tests indicated that certain combinations of pulse duration and interval could lead to unstable discharges. The selected parameter ranges allowed continuous machining without short-circuit pulses and ensured repeatable surface roughness values. The choice of ranges was also guided by the literature discussed in the Introduction section. Table 3 presents a summary of the electrical parameters and processing conditions adopted for the experimental tests.

**Table 3:** Machining conditions.

Parameter	Value
Workpiece	Hastelloy C-22
Electrode material	S-180 (10 $\upmu$m) and AF-5 (1 $\upmu$m)
Discharge time $t_{on}$ ( $\upmu$s)	8–55
Discharge current $I_c$ (A)	1.7–5
Time interval between pulses $t_{off}$ ( $\upmu$s)	6–75
Open voltage $U_0$ (V)	230
Discharge voltage $U_c$ (V)	26
Machining depth $a_p$ (mm)	0.5
Tool polarity	Negative (tool electrode–cathode, workpiece–anode)
Dielectric	EDM fluid 108 MP-SE

An experimental study was conducted to examine how the discharge current (${I}_{\hbox {c}}$), discharge time (${t}_{\hbox {on}}$), and time interval (${t}_{\hbox {off}}$) influence the surface roughness (*Sa, Sz, St, Sds, Sdr, Sfd*) of Hastelloy C-22 after electrical discharge machining (EDM) with a negative polarity setup. The experimental research was based on Hartley’s experimental design, incorporating three input variables each at five different levels. The values of the coded variables from the experimental design, along with their corresponding real variables, are presented in Table 4.

**Table 4:** Established the process conditions and their corresponding settings.

Levels	Discharge time ton ( $\upmu$s)	Discharge current Ic (A)	Time interval toff ( $\upmu$s)
−1.68	8	1.7	6
−1	17	2.7	19
0	30	3.8	37
1	41	4	51
1.68	55	5	75

Surface roughness measurements were performed using a Taylor Hobson FORM TALYSURF Series 2 scan profilometer. The instrument used for measurement provides a resolution of 0.6 nm. The radius of curvature of the probe tip was 2 $\upmu$m. For each sample, a sampling area of 4 mm^2^ was measured. A discretisation step of 10  $\upmu$m was applied along the Y-axis direction. The number of measurement points along the X-axis was 5,000.

For a comprehensive analysis of the surface topography, it was decided to determine the spatial roughness parameters, which provide additional information regarding the complexity of the surface stereometry (according to EUR 15778 EN standard). The parameters of surface roughness examined were:

- *Sa* - arithmetic mean roughness. The average of the absolute deviations of the surface height values from the mean plane over the entire measured area,
- *Sz* – it represents the average of the five highest peaks and the five deepest valleys within a sampling area,
- *St* - total height of the surface. The sum of the largest peak height and the deepest valley depth within the measurement area,
- *Sds* - density of summits. The number of local peaks (summits) per unit area,
- *Sdr* - developed surface area ratio. The ratio of the actual 3D surface area to the projected (flat) area, expressed as a percentage,
- *Sfd* - surface fractal dimension.

## Results and discussion

### Analysis of surface topography properties

**Fig. 2:**
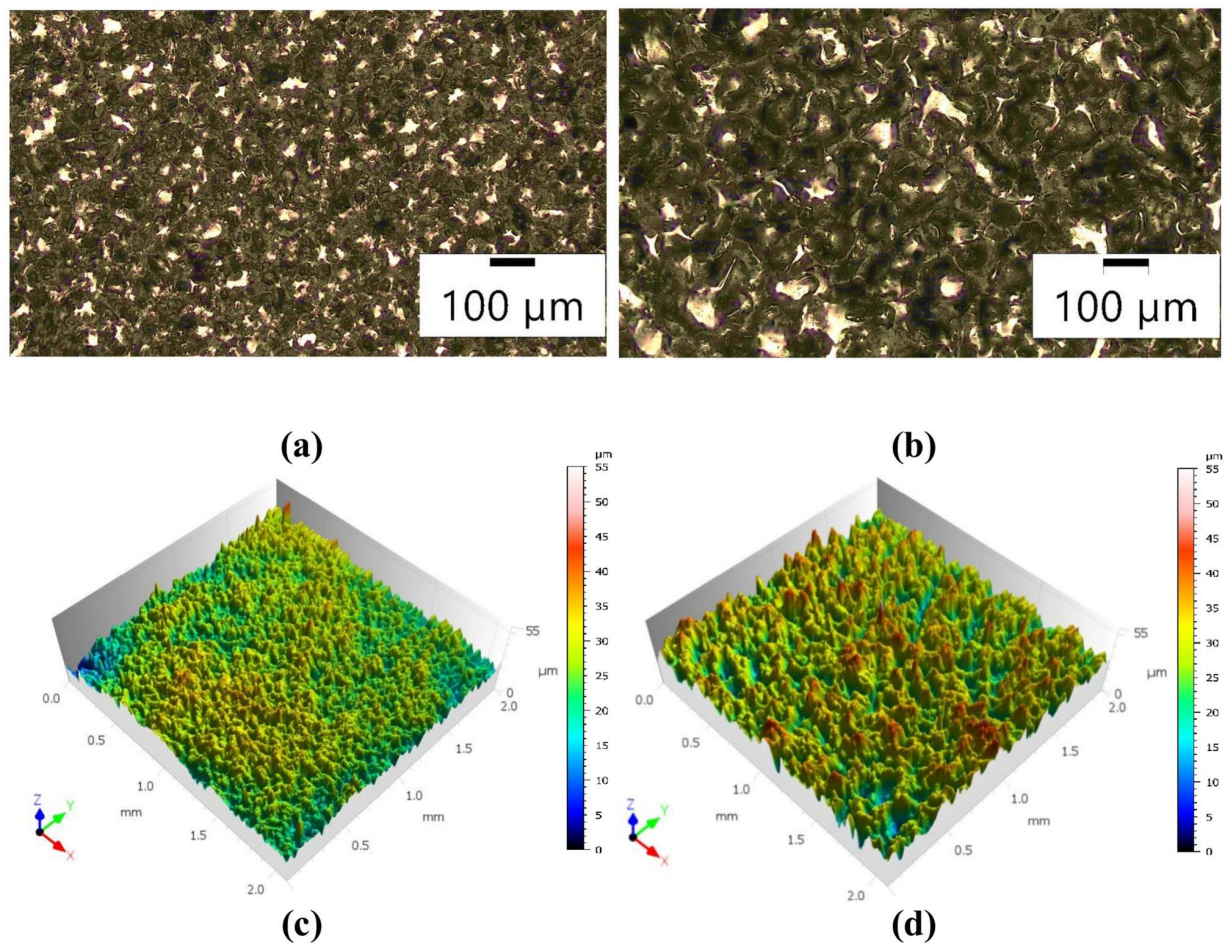
Surface morphology after EDM with negative polarity using AF-5 graphite electrode and the following parameters: ${t}_{\hbox {on}}$ = 30  $\upmu$s,* t*_off_ = 37  $\upmu$s. (**a**) and (**b**) optical microscope images at different current intensities: (**a**) ${I}_{\hbox {c}}$= 1.7 A, (**b**) ${I}_{\hbox {c}}$ = 5 A; (**c**) and (**d**) stereoscopic profilometer images showing surface topography corresponding to (**a**) and (**b**), respectively.

In electrical discharge machining, material removal is achieved through electrical discharges occurring between the cathode and anode. These discharges generate a plasma channel that melts and vaporises a volume of the workpiece material, resulting in the formation of a crater on the machined surface. The surface after electrical discharge machining is formed by the superposition of individual electrical discharge traces and is characterised by high isotropy and lacks a clear directional pattern (Fig. 2).

**Fig. 3:**
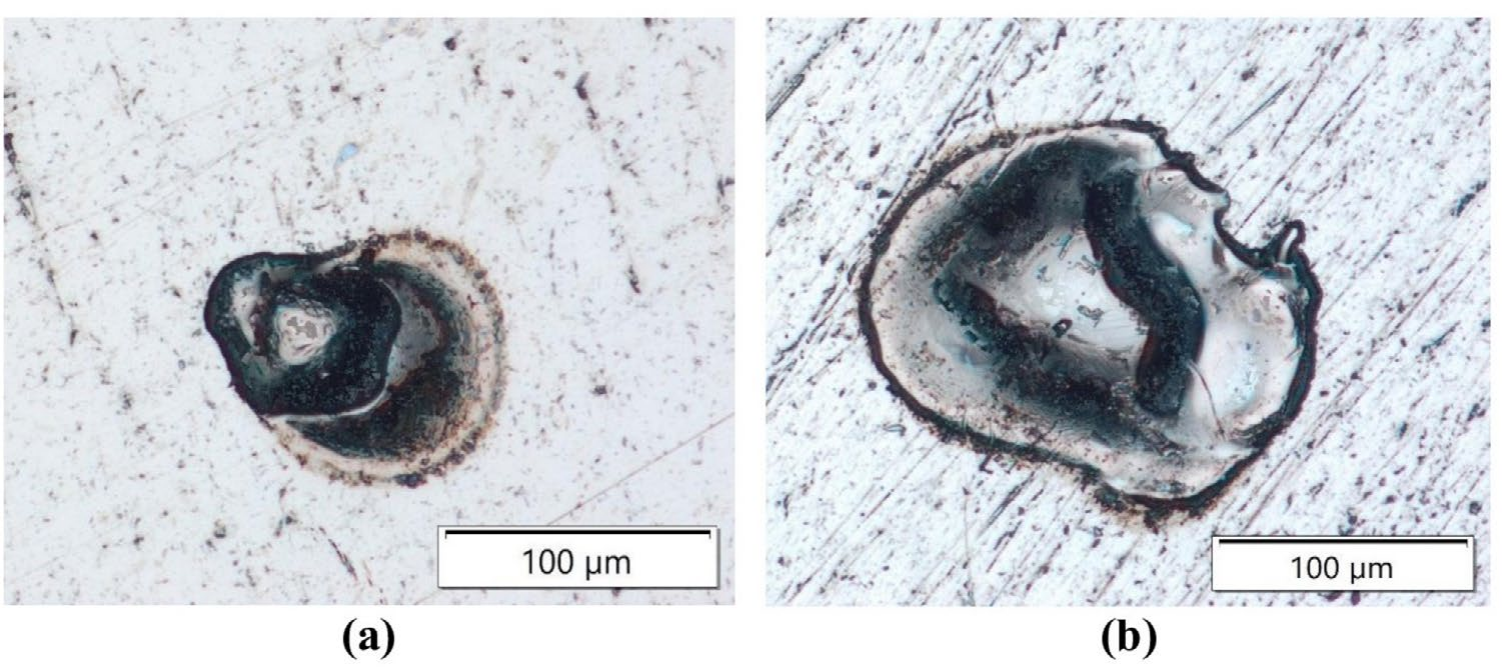
Single craters on the machined surface for AF-5 electrode and parameter values: *Uo* = 220 V, *U*_c_ = 25 V, ${t}_{\hbox {on}}$ = 31  $\upmu$s, ${t}_{\hbox {off}}$ = 37  $\upmu$s, a) ${I}_{\hbox {c}}$ = 1.7 A, b) ${I}_{\hbox {c}}$ = 5 A.

The distribution of craters on the machined surface is random. The morphology and dimensions of the craters are influenced by the electrical settings and the material characteristics of the graphite electrode. Following electro-erosion processing, the surface exhibits a layer of dark residue composed of erosion by-products, carbon resulting from dielectric pyrolysis, and detached grains originating from the graphite electrode. The state of the surface texture exerts a profound influence on the functional performance and tribological characteristics of the workpiece. Based on the conducted research, it was observed that the primary parameters influencing surface roughness after EDM machining are the current intensity and the electrical discharge duration. An increase in current intensity leads to an expansion of the plasma channel diameter and, consequently, an increase in crater volume. This is evidenced by the traces of individual electrical discharges observed on the machined surface (Fig. 3). Specifically, during machining at a current of ${I}_{\hbox {c}}$ = 5A, the volume of the eroded crater was nearly three times larger than that formed at ${I}_{\hbox {c}}$ = 1.7A. In negative polarity, the outflows are discontinuous and usually concentrated in a particular area in the immediate vicinity of the crater. At higher current levels, the craters exhibited more irregular shapes.

The physical phenomena inherent to the EDM process result in the removal of graphite electrode material during electrical discharges. Material loss is particularly significant for large-grain graphite electrodes. This process contributes to increased electrode wear and may also induce short-circuit pulses or electrical discharge dispersion^39^.

The surface chemical composition of selected samples was analysed using a Hitachi SU 3500 scanning electron microscope equipped with EDS. Spectroscopic analysis revealed the presence of carbon originating from the graphite electrode and, to some extent, from dielectric pyrolysis on the machined surface. During the recrystallisation process, carbon diffuses into the melted volume of the material. Elements such as nickel, chromium, molybdenum, iron, and manganese are derived from the native material of the sample.

**Fig. 4:**
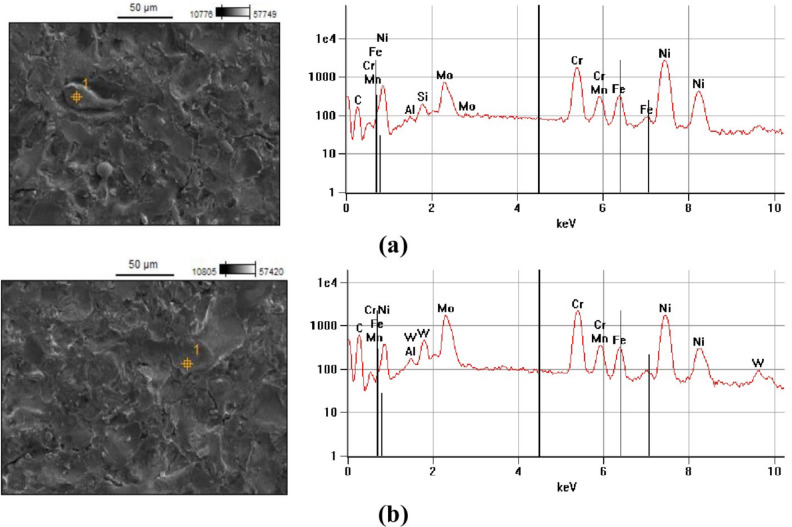
Surface EDS spectrum of the sample for negative polarity, processing parameters ${t}_{\hbox {on}}$ = 30  $\upmu$s,${t}_{\hbox {off}}$ = 37  $\upmu$s, ${I}_{\hbox {c }}$= 1.7 A, and graphite electrodes: (**a**) AF-5, (**b**) S-180.

For experiment number 9 (${t}_{\hbox {on}}$ = 30  $\upmu$s,* t*_off_ = 37  $\upmu$s, ${I}_{\hbox {c}}$= 1.7 A), which exhibited the highest level of tool electrode wear, the sample surface after machining with the AF-5 fine-grained electrode contained 7.6% carbon, as shown in Fig. 4. Conversely, using the coarse-grained S-180 graphite resulted in a carbon content twice as high, reaching 15.23%. Analysis of the EDS spectra from the electrode surfaces indicated that an increase in the electric discharge time causes an increase in the diffusion of Hastelloy C-22 alloy elements onto the tool surface.

**Fig. 5:**
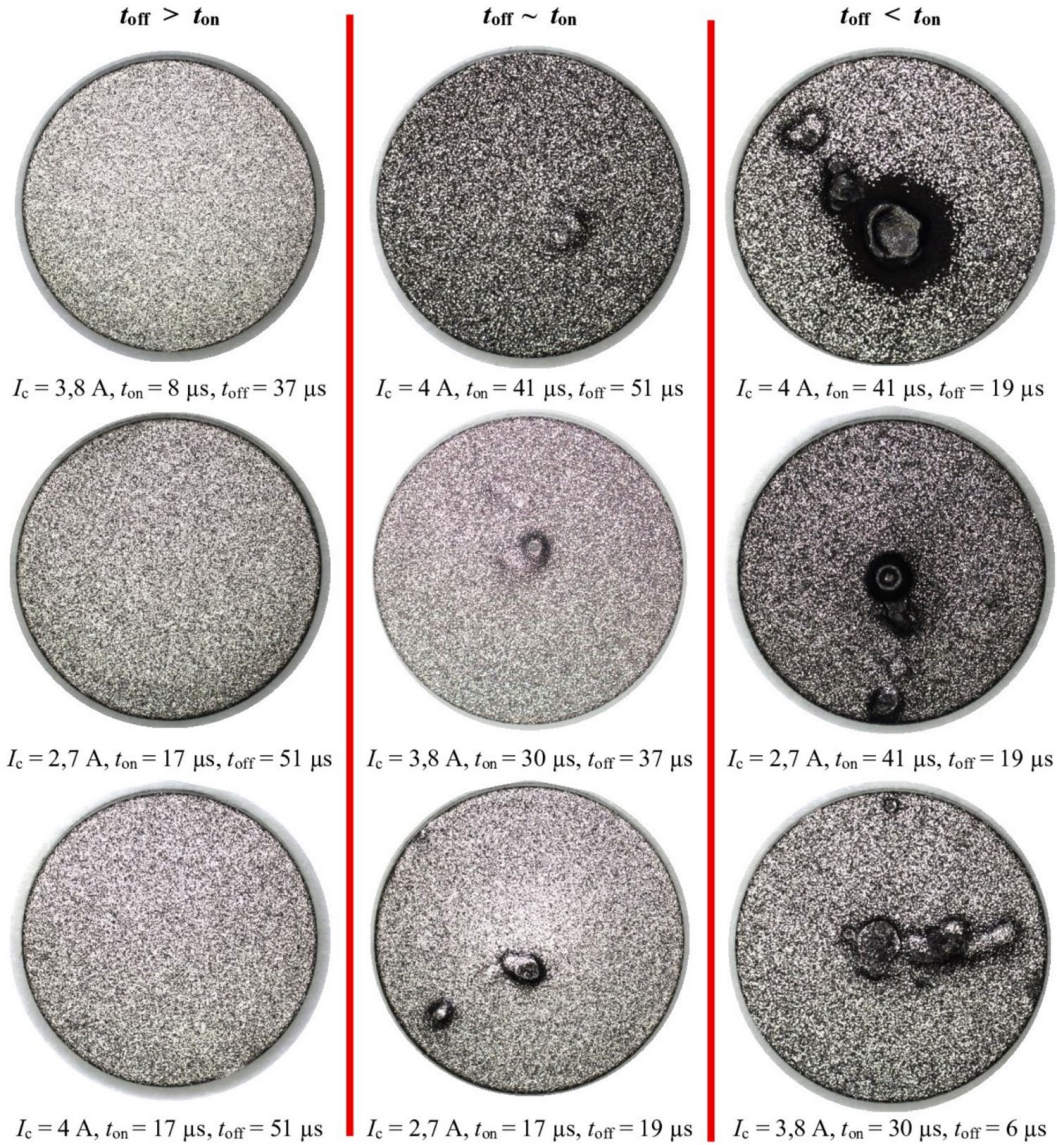
Visualisation of the external surface texture after EDM with the S-180 electrode for the cases: ${t}_{\hbox {off}} > {t}_{\hbox {on}}, {t}_{\hbox {off}} ~ {t}_{\hbox {on}}, {t}_{\hbox {off}} < {t}_{\hbox {on}}$.

**Fig. 6:**
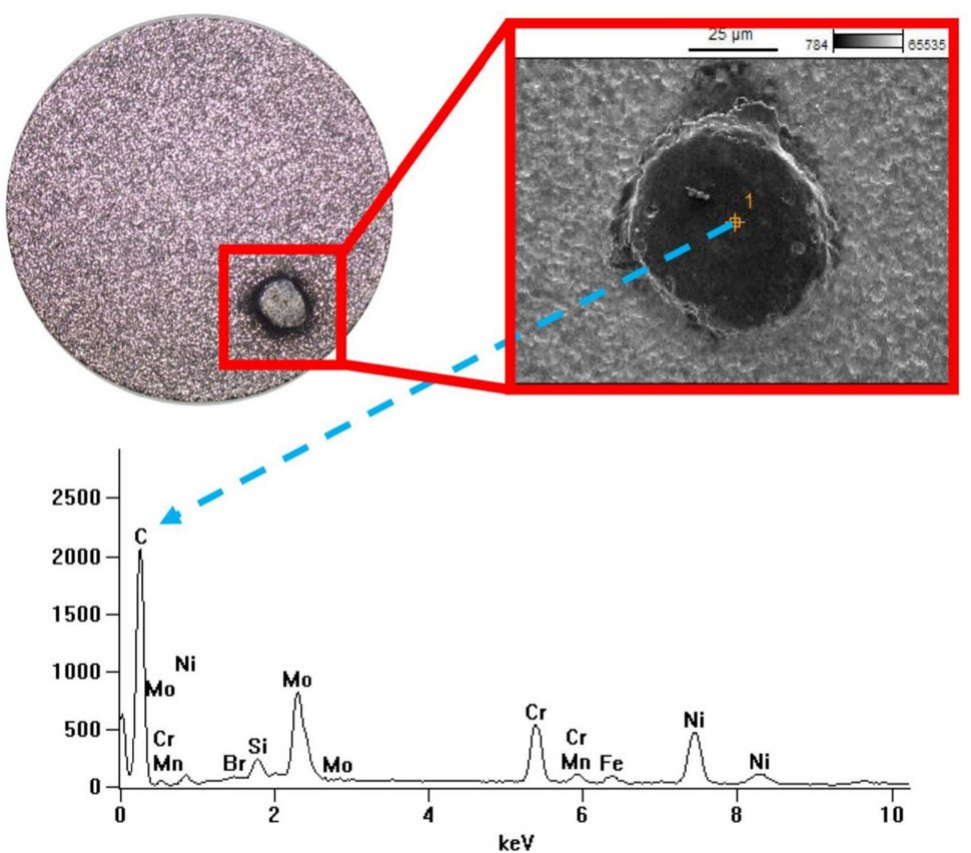
Analysis of the chemical composition and surface defect of the sample after AF-5 electrode machining with parameters: *I*_c_ = 5 A, ${t}_{\hbox {on}}$ = 30  $\upmu$s, ${t}_{\hbox {off}}$ = 37  $\upmu$s.

Following electrical discharge machining, deposits and burn marks were commonly observed on the sample surface (Fig. 5). These defects appeared when the electrical discharge duration (${t}_{\hbox {on}}$) exceeded the time interval (${t}_{\hbox {off}}$). Analysis of the EDS spectrum from a representative burn area revealed a predominant presence of carbon elements (Fig. 6). Due to the intensive wear of the tool electrode with negative polarity, detached graphite particles tend to accumulate in the region with the highest electric field intensity between the cathode and anode. An excess of graphite grains in the gap contributes to irregular electrical discharges and short-circuit pulses, which lead to localised burns on the machined surface. A free-kinematics EDM model, in which the tool electrode engaged only its frontal surface, was employed to minimise the influence of varying machining conditions with increasing machined depth. No additional gap flushing was applied; although this could result in local debris accumulation within the sparking gap and point-like surface burns, it ensured repeatable and well-controlled experimental conditions. Increasing the time interval between pulses (${t}_{\hbox {off}}$) reduces the occurrence of burns by more effectively removing graphite debris and erosion products from the sparking gap, thereby promoting more stable electrical discharges.

### Analysis of surface roughness parameters

Tables 5 and 6 present the results of roughness measurements of samples after machining with the S-180 and AF-5 tool electrodes. The arithmetic mean deviation of surface roughness (*Sa* parameter) ranges from 2.07 to 5.23  $\upmu$m. These values are characteristic of surface roughness obtained under semi-finishing and finishing EDM conditions. The *Sa* parameter is primarily influenced by the current intensity (*I*_c_) and the electrical discharge duration (${t}_{\hbox {on}}$). An increase in both parameters increases the diameter and power of the plasma channel, which results in deeper penetration and erosion of a larger volume of material from the workpiece. Deep craters formed on the surface cause an increase in the height and longitudinal roughness parameters.

**Table 5:** Summary of surface roughness measurements of samples machined with the AF-5 electrode.

No.	Ic	ton	toff	Sa	Sz	St	Sds	Sfd	Sdr
	(A)	( $\upmu$s)	( $\upmu$s)	( $\upmu$m)	( $\upmu$m)	( $\upmu$m)	(pks/mm2)	(–)	(%)
1	2.7	17	19	3.57	32.5	35.4	724	2.4	3.43
2	2.7	17	51	2.55	27.7	32.5	777	2.47	3.31
3	2.7	41	19	3.76	37.3	55	749	2.4	4.43
4	2.7	41	51	3.56	38.3	47.6	754	2.41	4.3
5	4	17	19	3.02	27	33.6	466	2.32	4.52
6	4	17	51	2.98	29.3	37.5	663	2.47	4.49
7	4	41	19	4.54	45.1	59.7	536	2.4	5.66
8	4	41	51	4.61	44.7	52	568	2.41	5.83
9	1.7	30	37	2.92	25.6	51	1240	2.41	2.43
10	5	30	37	4.3	42.1	53.5	479	2.42	5.49
11	3.8	8	37	2.07	22.9	28.2	902	2.51	3.54
12	3.8	55	37	5.08	48.5	54.8	378	2.39	6.35
13	3.8	30	6	4.21	38.3	47.5	597	2.42	4.8
14	3.8	30	75	3.73	37.8	50	587	2.42	5.04
15	3.8	30	37	4.37	41.1	55.8	579	2.42	5.25
16	3.8	30	37	4.37	43.6	55.7	611	2.41	5.3

**Table 6:** Summary of surface roughness measurements of samples machined with the S-180 electrode.

No.	Ic	ton	toff	Sa	Sz	St	Sds	Sfd	Sdr
	(A)	( $\upmu$s)	( $\upmu$s)	( $\upmu$m)	( $\upmu$m)	( $\upmu$m)	(pks/mm2)	(–)	(%)
1	2.7	17	19	3.29	26.1	42.3	867	2.42	2.99
2	2.7	17	51	2.47	22.6	26.8	583	2.36	2.84
3	2.7	41	19	3.57	31.4	38.2	826	2.42	4.02
4	2.7	41	51	3.24	30.1	36.6	778	2.42	3.92
5	4	17	19	2.83	28.4	33.4	569	2.46	3.87
6	4	17	51	3.01	31.6	35.5	654	2.46	4.16
7	4	41	19	4.33	43.2	49.8	567	2.38	4.59
8	4	41	51	4.44	44.7	53.7	563	2.4	5.46
9	1.7	30	37	3.33	21.4	30.1	1098	2.4	2.44
10	5	30	37	5.23	32.4	45.8	482	2.4	5.97
11	3.8	8	37	2.47	25.6	31.4	769	2.45	3.46
12	3.8	55	37	4.71	44.3	51.8	569	2.4	5.42
13	3.8	30	6	4.13	45.2	52.3	598	2.4	5.01
14	3.8	30	75	3.69	40.5	50.9	525	2.41	4.8
15	3.8	30	37	4.4	38.1	52.7	552	2.42	4.97
16	3.8	30	37	3.66	38.1	42.8	556	2.43	4.86

The *Sa* parameter values are similar for both the fine-grained (AF-5) and coarse-grained (S-180) electrodes. This indicates that the primary factor determining the *Sa* roughness value in negative polarity is the electrical parameters (discharge current and discharge time), and to a lesser extent, the graphite electrode grain size. Electrons emitted from the cathode surface in negative polarity transfer greater thermal energy to the workpiece, resulting in the removal of a larger volume of material from the anode surface and consequently producing higher surface roughness. The grain size has minimal impact on the surface layer condition, as the thermal energy distribution during machining in reverse polarity predominantly governs the surface finish. A comparative analysis of the *Sz* and *St* parameters can provide insights into the stability of electrical discharges. In most cases, these parameters exhibit similar values, indicating stable electrical discharges within the adopted experimental setup and a low proportion of random peaks and valleys in surface irregularities. The correlation between the discharge time (${t}_{\hbox {on}}$) and the time interval between pulses (${t}_{\hbox {off}}$) significantly influences the values of these parameters. This effect arises from the fact that graphite grains detached during electrical discharges are removed from the gap during the pause between pulses. Extending the time interval (${t}_{\hbox {on}}$), which facilitates more effective removal of machining debris and graphite grains, results in more intense electrical discharges within the gap. Consequently, this leads to deeper surface penetrations and higher peaks in surface irregularities. The AF-5 electrode, characterised by a higher apparent density and smaller grain size compared to the S-180 electrode, exhibits lower wear. The smaller grains are easier to remove from the inter-electrode gap than the coarser graphite grains. As a result, more pronounced differences between the *Sz* and *St* parameters are observed on surfaces machined with the AF-5 fine-grained graphite electrode. The easier removal of machining by-products in this case promotes more intense electrical discharges, producing deeper penetrations and more irregular surface morphology.

The shape and complexity of surface roughness peaks can be described using the density of summits (*Sds*), the relative surface area development (*Sdr*), and the fractal dimension (*Sfd*), which quantifies surface similarity. The hybrid parameter *Sds* reflects the number of peaks within a single sampling area and indirectly indicates the spacing between successive roughness peaks. A higher *Sds* value suggests a greater number of peaks within the same area. Variations in the number of roughness peaks with similar heights may indicate that the resulting craters have comparable depths but different diameters. The *Sdr* parameter provides information about the actual contact area, which is crucial for surface bonding, adhesion, and coating processes. A detailed analysis of the combined *Sds* and *Sdr* parameters offers valuable insights into surface abrasion, coating suitability, and reflectivity.

Both *Sds* and *Sdr* parameters show a strong dependence on the current (*I*_c_) and the discharge time (${t}_{\hbox {on}}$). At lower discharge energy levels, the surface is characterised by a higher density of roughness peaks and less surface development. As the current (*I*_c_) and discharge time (${t}_{\hbox {on}}$) increase, the surface becomes smoother. No significant correlation was observed between the grain size of the graphite electrode and the hybrid roughness parameters *Sds* and *Sdr*; these parameters are primarily influenced by the current-voltage characteristics of the discharge process.

The fractal dimension (*Sfd*) varies within a narrow range, regardless of the EDM parameters. This suggests that the complexity of the surface texture is relatively unaffected by the process parameters and is more characteristic of the entire EDM machining procedure. This finding aligns with the results reported by Żak^42,43^, who indicated that the fractal dimension can serve as a parameter for assessing the similarity of surfaces obtained in different machining operations.

### Statistical models od *Sa*, *Sz*, *St*, *Sds*, *Sdr*, and *Sfd*

The research employed Hartley’s experimental design methodology, incorporating five levels and three independent electrical variables: current intensity (*I*_c_), discharge duration (${t}_{\hbox {on}}$), and time interval (${t}_{\hbox {off}}$). To verify the consistency of the outcomes, two replicates were performed at the central level of the experimental matrix. According to the experimental framework, a total of 16 experiments were conducted, each with distinct combinations of electrical parameters. All experimental procedures were performed under consistent machining conditions using two types of graphite electrodes: AF-5 and S-180.

The experimental data served as the basis for developing mathematical models expressed as second-degree polynomial regressions, which describe the influence of the investigated process parameters and the graphite electrode grain size on surface roughness parameters such as *Sa*, *Sz*, *St*, *Sds*, *Sdr*, and *Sfd*. These regression models were determined utilising the STATISTICA 13.3 software, employing a step-back regression approach to identify the most significant variables.

Empirical models were developed using second-degree polynomial equations to describe the impact of selected processing parameters on surface roughness characteristics. The goodness-of-fit of each regression model to the experimental data was quantified using the correlation coefficient, *R*. This coefficient indicates the strength of the statistical relationship between the input variables of the process and the resulting surface roughness parameters of the obtained regression equation. For each model, the value of *R* was calculated, with values approaching one signifying a stronger correlation and better representation of the variability within the studied data.

The significance of the regression models was assessed using the Fisher–Snedecor *F*-test, where the F-statistic was compared against the critical *F*-value (*F*_kr_). A model was considered statistically significant if the ratio *F*/*F*_kr_ was greater than or equal to one at a significance level of *p* = 0.05.

Furthermore, the significance of individual regression coefficients was evaluated using Student’s *t*-test, where the calculated *t*-value was contrasted with the critical value of *t*_kr_, at the same significance level (*p* = 0.05). Regression terms were considered statistically significant if ${t} > {t}_{\hbox {kr}}$.

This study employed response surface methodology (RSM) to develop predictive regression models for the electrical discharge machining with graphite electrodes of various grain sizes. The resulting equations, which describe the relationships between the process parameters and surface roughness (*Sa, Sz, St, Sds, Sdr*, and *Sfd*), demonstrate high correlation coefficients (*R*). In all cases, the *F*-value divided by the *F*-critical value (*F*/*F*_kr_) markedly exceeds unity, indicating the statistical significance of the models. The standard errors associated with the estimates are maintained at acceptably low levels across all equations, confirming their reliability. A comprehensive summary of the regression analysis, including key statistical metrics, is provided in Table 7.

**Table 7:** Regression summary.

POCO graphite	Parameter	R	F/Fkr
S-180	Sa	0.93	2.7
S-180	Sz	0.84	1.8
S-180	St	0.82	1.4
S-180	Sds	0.92	2.6
S-180	Sfd	0.90	1.5
S-180	Sdr	0.96	4
AF-5	Sa	0.91	4.5
AF-5	Sz	0.93	4.5
AF-5	St	0.92	2
AF-5	Sds	0.90	2.1
AF-5	Sfd	0.80	1.1
AF-5	Sdr	0.99	23

**Fig. 7:**
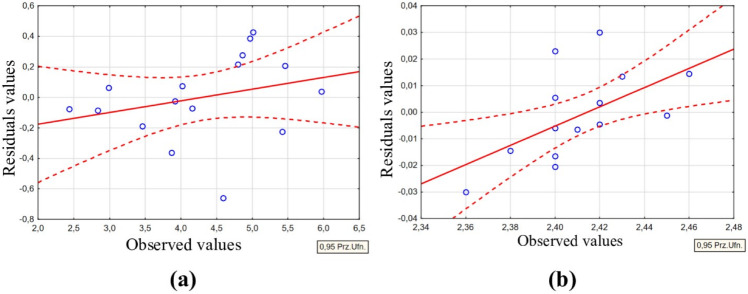
Residuals as a function of observed values for the equation and tool: (**a**) *Sdr*, S-180 electrode; (**b**) *Sfd*, AF-5 electrode.

For every regression model, residuals were plotted against the observed data points. An example of such residual plots for the *Sfd *and* Sdr *parameters is presented in Fig. 7. The analysis enables us to evaluate the accuracy of fitting experimental data to the established regression equation. The developed models demonstrate a low level of deviation between the observed and estimated values, indicating a good fit. These findings affirm the reliability of the developed mathematical models and underscore the substantial influence of the selected input parameters on the output results.

After statistically insignificant terms were eliminated, definitive quadratic regression equations were developed for each surface roughness parameter. A step-back regression procedure was applied, in which all linear, quadratic, and interaction terms were initially considered. Only statistically significant terms (p < 0.05) were retained. Consequently, quadratic terms for pulse duration (${t}_{\hbox {on}}^{2}$) and discharge current (${I}_{\hbox {c}}^{2}$) appear only in some models, while in others they were excluded due to their lack of statistical significance. Similarly, interaction terms were included only when they contributed significantly to explaining the variability in the surface roughness parameters. This approach ensures that the presented regression equations accurately describe the influence of the EDM parameters while avoiding overfitting.

Following the removal of non-essential terms from the equation, a definitive quadratic polynomial function was developed to characterise the effect of machining parameters on the surface roughness parameters. Regression equations describing the influence of EDM machining parameters on surface roughness parameters utilising the S-180 coarse-grained electrode (1-6):

1 $$\begin{aligned} Sa&= 4.66 - 1.6 \cdot I_c + 0.3 \cdot I_c^2 + 0.01 \cdot I_c \cdot t_{on} \quad \end{aligned}$$

2 $$\begin{aligned} Sz&= 23 + 0.12 \cdot I_c \cdot t_{on} \quad \end{aligned}$$

3 $$\begin{aligned} St&= 30 + 0.13 \cdot I_c \cdot t_{on} \quad \end{aligned}$$

4 $$\begin{aligned} Sds&= 1674 - 455 \cdot I_c + 62 \cdot I_c^2 + 0.2 \cdot t_{on}^2 - 4.3 \cdot I_c \cdot t_{on} \quad \end{aligned}$$

5 $$\begin{aligned} Sfd&= 2.3 + 0.06 \cdot I_c + 0.007 \cdot t_{on} - 0.005 \cdot t_{off} - 0.003 \cdot I_c \cdot t_{on} + 0.0009 \cdot I_c \cdot t_{off} + 0.00006 \cdot t_{on} \cdot t_{off} \quad \end{aligned}$$

6 $$\begin{aligned} Sdr&= -0.5 + I_c + 0.04 \cdot t_{on} \end{aligned}$$

Regression equations describing the influence of EDM machining parameters on surface roughness parameters utilising the AF-5 fine-grained electrode (7-12):

7 $$\begin{aligned} Sa&= 2.5 - 0.1 \cdot I_c^2 - 0.001 \cdot t_{on}^2 + 0.04 \cdot I_c \cdot t_{on} \quad \end{aligned}$$

8 $$\begin{aligned} Sz&= 24 + 0.13 \cdot I_c \cdot t_{on} \quad \end{aligned}$$

9 $$\begin{aligned} St&= 9 + 2 \cdot t_{on} - 0.02 \cdot t_{on}^2 \quad \end{aligned}$$

10 $$\begin{aligned} Sds&= 2189 - 635 \cdot I_c + 74 \cdot I_c^2 - 1.6 \cdot I_c \cdot t_{on} \quad \end{aligned}$$

11 $$\begin{aligned} Sfd&= 2 + 0.09 \cdot I_c + 0.009 \cdot t_{on} - 0.003 \cdot I_c \cdot t_{on} \quad \end{aligned}$$

12 $$\begin{aligned} Sdr&= -1.6 + 2.3 \cdot I_c - 0.3 \cdot I_c^2 + 0.01 \cdot I_c \cdot t_{on} \quad \end{aligned}$$

**Fig. 8:**
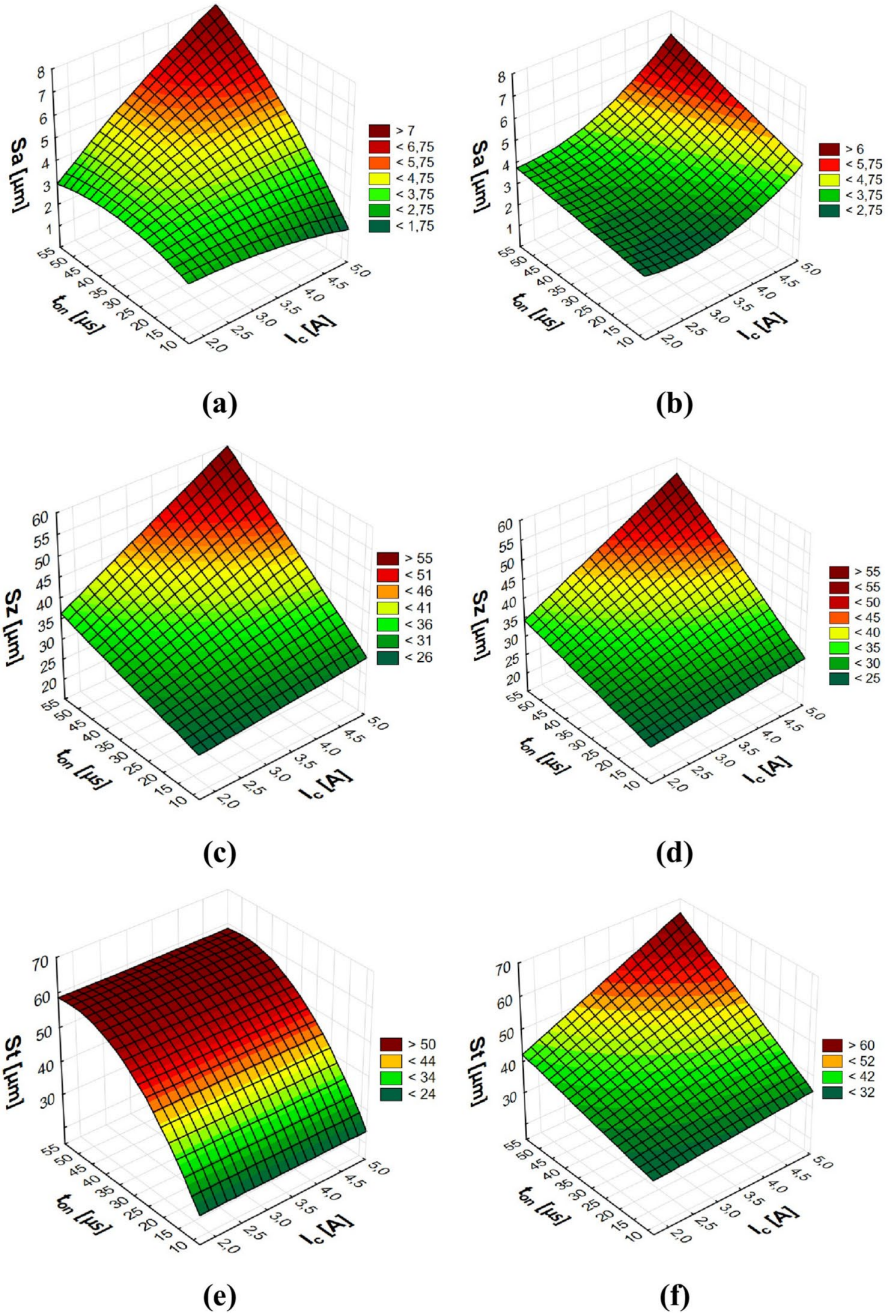
Graphical illustration of the regression equation for the* Sa, Sz *and *St* parameter after EDM with the electrode: (**a**), (**c**), (**e**) AF-5, and (**b**), (**d**), (**f**) S-180.

**Fig. 9:**
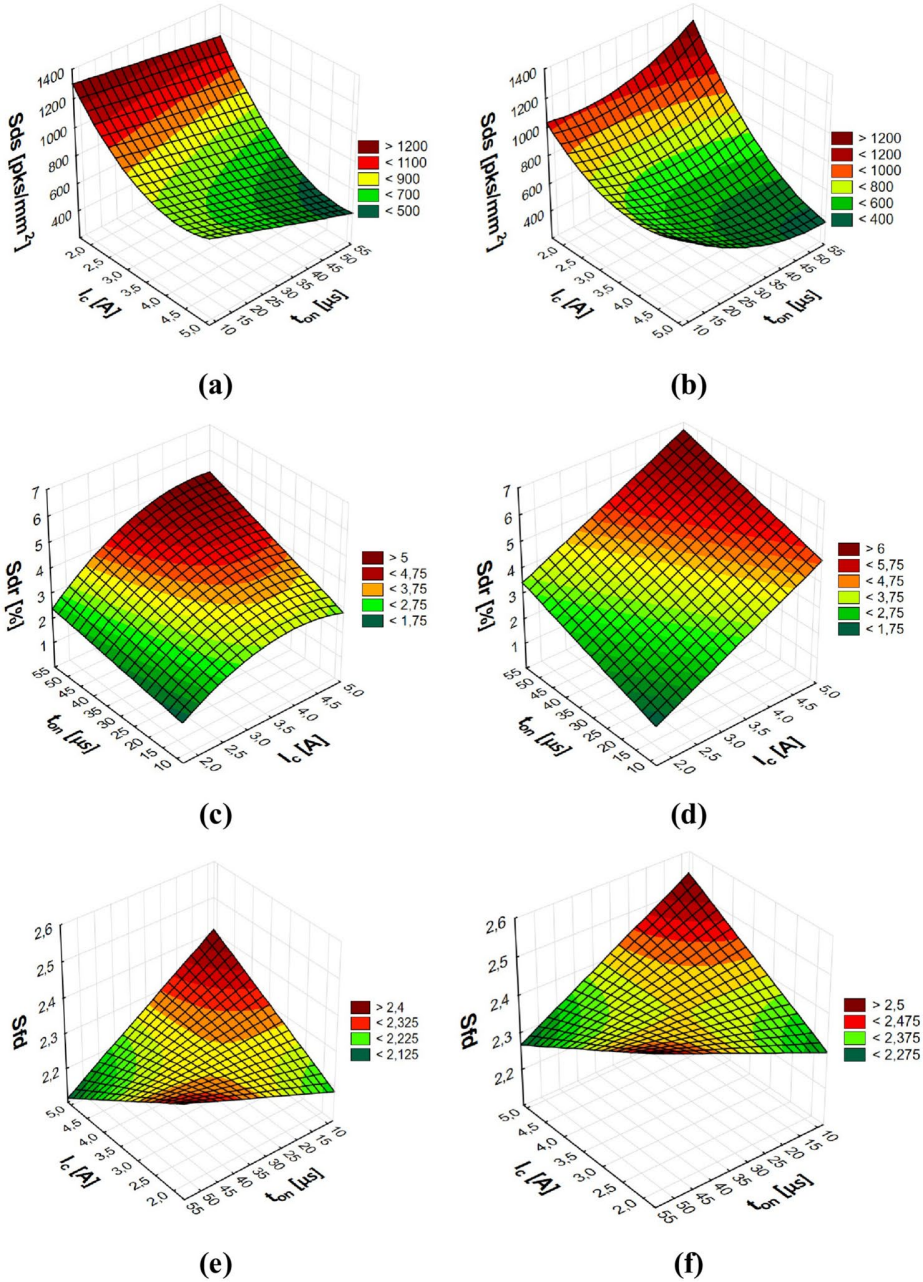
Graphical illustration of the regression equation for the *Sds, Sdr* and *Sfd* parameter after EDM with the electrode: (**a**), (**c**), (**e**) AF-5, and (**b**), (**d**), (**f**) S-180.

The regression models derived from the analysis are visually depicted in Figs. 8, 9.

The parameters describing surface roughness are strongly correlated with the energy associated with individual electrical discharges. These parameters are directly affected by the amount of material removed during each discharge event. An escalation in discharge energy results in a higher heat flux density within the plasma channel, which in turn enhances the melting and vaporisation of the material. This process produces a larger crater, both in depth and width, thereby elevating the roughness amplitude. As a result, these changes significantly impact the *Sa*, *Sz* and *St* roughness parameters.

The time interval (${t}_{\hbox {off}}$) has no statistical effect on the surface texture after EDM. This parameter does not affect the volume of material removed from the workpiece. The duration of the time interval influences both the machining efficiency and the stability of the process. In principle, reducing the interval duration can enhance machining throughput; however, if the interval is excessively brief, the expelled material from the workpiece may not be effectively removed by the dielectric flow, leading to incomplete deionisation of the fluid. It can lead to unstable electrical discharges during subsequent sparks and the formation of defects and burns on the machined surface.

At low current levels, the surface roughness remained largely unaffected despite variations in pulse duration. As the discharge duration (${t}_{\hbox {on}}$) extends, the diameter of the plasma channel expands, while the energy density decreases, reducing the volume of melted material, which influences the surface roughness after machining. At the same time, the low-energy plasma channel is cooled by the dielectric liquid. Furthermore, extending the discharge time at high current values increases the plasma channel diameter and reduces the thermal energy density. Craters formed under such processing conditions are characterised by larger dimensions and depths, which increase surface roughness.

The morphology of roughness vertices can be characterised by the density of the topographical parameter *Sds*. This parameter is influenced by both the magnitude of the discharge current (*I*_c_) and the discharge time (${t}_{\hbox {on}}$). When higher levels of discharge current and extended discharge times are applied, the resulting lowest density of tops *(Sds*) and the highest value of the *Sa* roughness parameter. It indicates that the resulting craters formed by electrical discharges have the greatest diameters and depths. The surface texture arises from the overlapping of individual discharge traces. An increase in current (*I*_c_) and discharge duration (${t}_{\hbox {on}}$) enhances the plasma channel’s diameter and energy, which in turn produces a rougher surface profile with larger spacing between the vertices. Conversely, shorter discharge times combined with lower current levels result in a surface characterised by a higher density of vertices, reflecting a finer and more densely packed surface topography.

## Conclusions

The experimental investigation centred on assessing how the grain size of the graphite electrodes (specifically AF-5 and EDM-180) and the electrical process parameters influence the surface roughness of Hastelloy C-22 following electrical discharge machining with negative polarity. The effects of both grain size and electrical variables on surface roughness characteristics were examined and quantified. Surface texture was evaluated through detailed observations, and surface roughness parameters–including *Sa, Sz, St, Sds, Sdr*, and *Sfd*–were measured. In the concluding part of the study, mathematical models were formulated to illustrate the relationship between the process parameters and surface roughness outcomes. Based on the experimental data, the following conclusions were drawn:

1. The grain size of the graphite tool electrode has an insignificant influence on the surface roughness following EDM processing with negative polarity.
2. The surface roughness of the Hastelloy C-22 alloy after EDM was mainly determined by the electrical parameters of the process.
3. The height roughness parameters - *Sa, Sz, St*, and the hybrid roughness parameters - *Sds, Sdr, Sfd* were primarily influenced by the current intensity (*I*_c_) and the electric discharge time (${t}_{\hbox {on}}$).
4. At lower values of electric discharge energy, the surface is characterised by a higher density of unevenness peaks (*Sds*) and reduced surface development (*Sdr*).
5. The surface fractal dimension (*Sfd*) varies within a very narrow range with varying EDM parameters. This parameter can be used to determine the similarity of surfaces obtained in different machining operations.
6. The increase in current intensity (*I*_c_) and electric discharge time (${t}_{\hbox {on}}$) results in a greater material removal volume, leading to the formation of a larger crater with increased diameter and depth, consequently elevating the surface roughness.
7. Extending the time interval ${t}_{\hbox {off}}$ reduces the likelihood of burns due to more effective cleaning of the sparking gap from graphite grains and erosion products.
8. Detached graphite tool particles can affect the variation in current magnitude and the discharge voltage within the sparking gap. The likelihood of short-circuit pulses increases when the gap becomes significantly contaminated with machining residues and sizable graphite grains.
9. Graphite tool particles tend to accumulate within the sparking gap, particularly at the location of maximum electric field between the cathode and anode. This accumulation can lead to short-circuiting events, generating localised thermal damage (burning) and compromising the integrity of the machined surface.
10. The predictive models created for the electrical discharge machining (EDM) of Hastelloy C-22 with graphite electrodes can be utilised to develop process parameter tables in advanced erosion equipment.

## Data Availability

The datasets generated and analysed during the current study are available from the corresponding author on reasonable request.
